# Lognormality in Turbulence Energy Spectra

**DOI:** 10.3390/e22060669

**Published:** 2020-06-17

**Authors:** Taewoo Lee

**Affiliations:** Mechanical and Aerospace Engineering, SEMTE, Arizona State University, Tempe, AZ 85287, USA; attwl@asu.edu

**Keywords:** maximum entropy principle, information theory

## Abstract

The maximum entropy principle states that the energy distribution will tend toward a state of maximum entropy under the physical constraints, such as the zero energy at the boundaries and a fixed total energy content. For the turbulence energy spectra, a distribution function that maximizes entropy with these physical constraints is a lognormal function due to its asymmetrical descent to zero energy at the boundary lengths scales. This distribution function agrees quite well with the experimental data over a wide range of energy and length scales. For turbulent flows, this approach is effective since the energy and length scales are determined primarily by the Reynolds number. The total turbulence kinetic energy will set the height of the distribution, while the ratio of length scales will determine the width. This makes it possible to reconstruct the power spectra using the Reynolds number as a parameter.

## 1. Introduction

**Figure 1:**
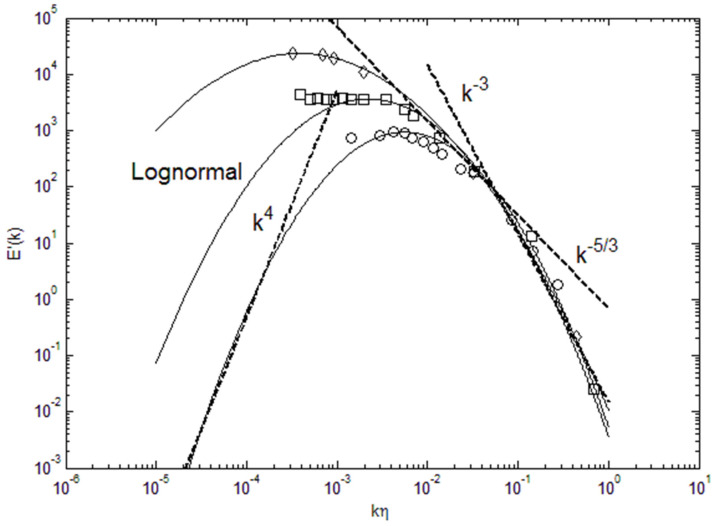
Lognormal energy spectra and various k^n^-scaling. The data are from Comte-Bellot and Corrsin (**circle**) [7], Champagne, et al. (**square**) [8] and Saddoughi and Veeravalli (**diamond**) [9].

The maximum entropy principle is very useful, in determining blackbody radiation spectra [1], energy distribution in particles [2], and in specifying drop size distributions [3], as some examples. This principle states that the energy distribution of particles will tend toward the state of maximum entropy under the given constraints of the physical system. Turbulence can be considered as a large ensemble of energetic eddies having a spectrum of energy and length scales. Due to the large size of the ensemble, it will come to an equilibrium state of maximum entropy under the constraints of zero energy at the boundary points, fixed total energy content and viscous dissipation. The total energy and the range of length scales that exist in the turbulent flow primarily depend on the Reynolds number. For example, the total turbulence kinetic energy contained in the energy spectrum will be specified by the initial mean velocity and length scale of the flow, in other words by the Reynolds number. In order to find some universal laws concerning the turbulence kinetic energy spectrum, some insights were provided through theoretical analyses [4,5,6]. In Figure 1, the scaling laws from some of these analyses are plotted, and compared with lognormal distribution and data. We can see that various scaling laws are tangent to the lognormal distribution in the wavenumber regions of their applicability. Notably, in the inertial subrange the Kolmogorov k^−5/3^ scaling will track the power spectra. We can see in Figure 1 that due to broadening of the energy spectrum with increasing total energy, or Reynolds number, there will be an increase in the wavenumber range where k^−5/3^ scaling will be nearly tangent to the spectrum. Additionally, the ascending part of the spectra at low wavenumbers (largest eddies) is tracked by k^4^-scaling [6], which is tangent to lognormal function at this range. We can see that k^n^-type of scaling will be tangent to lognormal at least at some point and range of the wavenumber space. However, the range of its applicability tends to be limited as tangent lines do not fully express the curved energy spectrum.

Lognormality occurs often in nature, including turbulence. Recently, lognormality in turbulence dissipation has been observed in large-scale flows [10]. Intermittency has been modelled as being lognormally distributed in space, but with some conceptual and mathematical inconsistencies that needed to be addressed [11]. Additionally, Mouri et al. [12] have noted on fluctuations of velocity and energy dissipation being lognormal in different flow geometries. However, the maximum entropy principle is directly applicable to energy distributions with intuitive and observable constraint parameters, in this case leading to lognormal turbulence energy spectra. Some studies were onto the use of the maximum entropy method in turbulence, but the applied constraints were too complicated and also the aim appears to have been in replicating the k^−5/3^ scaling [13,14], which is hard to achieve in typical maximum-entropy probability distribution functions (inverse exponential). If one looks at the data from a quiescent perspective, k^−n^ type of scaling is only partially applicable over a small subset of the spectrum, and the overall spectra tend to be parabolic in the logarithmic scale. 

In this work, we will validate this lognormality using experimental data over a wide range of Reynolds number. Key attributes of the energy spectra, the height and width, depend primarily on the Reynolds number, and possibly other easily observable parameters. This opens ways to reconstruct the power spectra, as the Reynolds number contains information concerning both the energy and the range of length scales that exist in the flow. There are some secondary parameters that are used in collapsing the power spectra [9], such as the dissipation and kinematic viscosity, and these may be used to fine-tune the lognormal function. However, the primary parameter for both the height and width of the lognormal spectra is just the Reynolds number, as will be shown.

## 2. The Maximum Entropy Principle

The maximum entropy principle states that the energy distribution will tend toward a state of maximum entropy (Shannon’s entropy) under the physical constraints, such as the zero energy at the boundaries and a fixed total energy content. For turbulence, the maximum entropy principle can be effective since the energy spectra will take on a distribution that achieves the maximum entropy, as constrained by the boundary conditions: The kinetic energy must be zero at the smallest dissipation scale (Kolmogorov scale), and also at the largest flow length scale (e.g., dimension of the object in the flow). The range of length scales, or the ratio of length scales from Kolmogorov (η) to the integral scales (*l*_e_), is known from η = *l*_e_Re_λ_^−3/2^ [6], where Re_λ_ is the Reynolds number based on the Taylor microscale (λ). Another important constraint is the total turbulent kinetic energy, fixed by the initial or external conditions.

In some cases, the distribution function can be derived algebraically [14]. Using the Lagrange multiplier method, the distribution function that maximizes the Shannon’s entropy (S=−ElnE) for turbulence energy can be derived as $E\left(k\right)=\frac{C_{1}}{k^{4}}exp\left\{-C_{2}u^{\prime 2}-C_{3}k^{2}u^{\prime 2}\right\}$, where C_1_, C_2_ are constants, to be determined from the constraints, and k the wavenumber. u′(k) is empirically input as (m-log(k)). The derivation is shown in the Appendix. This incidentally resembles the lognormal function, and has asymmetrical descent to zero energy at the boundary points. An alternate deductive method for arriving at the lognormal form is to test a sequence of standard distribution function (e.g., Gaussian, exponential, lognormal, etc.) with the maximum Shannon’s entropy that still obeys the physical constraints [2]. Then, the first simplest distribution that satisfy the constraints is the most likely one [2]. In this process, uniform, Gaussian and exponential distributions do not satisfy the asymmetric boundary conditions for turbulence energy spectra, thus pointing to the lognormal as the most likely distribution. Thus, it can be deduced that the distribution function that satisfies the boundary conditions is the lognormal function due to its asymmetric decay to zero at the boundary points. Energy spectra are asymmetrical because the descent toward zero energy occurs due to physical limit of the flow scale at the low wavenumber extreme, while viscous dissipation causes the approach toward zero at the high wavenumbers. For a similar reason, the drop size distributions in spray flows take on lognormal shape [15]. As noted above the width of the distribution can then be deduced from η = *l*_e_Re_λ_^−3/2^, while the height of the distribution is set by the total integrated turbulence kinetic energy, which is proportional to the mean velocity squared and the length scales of the flow. For example, atmospheric turbulence will have a very large total integrated energy and also the ratio of largest to the smallest (Kolmogorov) scales will be very large, both of which depend on the Reynolds number. Lognormal distribution has convenient parameterization aspects for these length scale effects, as shown later. 

## 3. Results and Discussion

**Figure 2:**
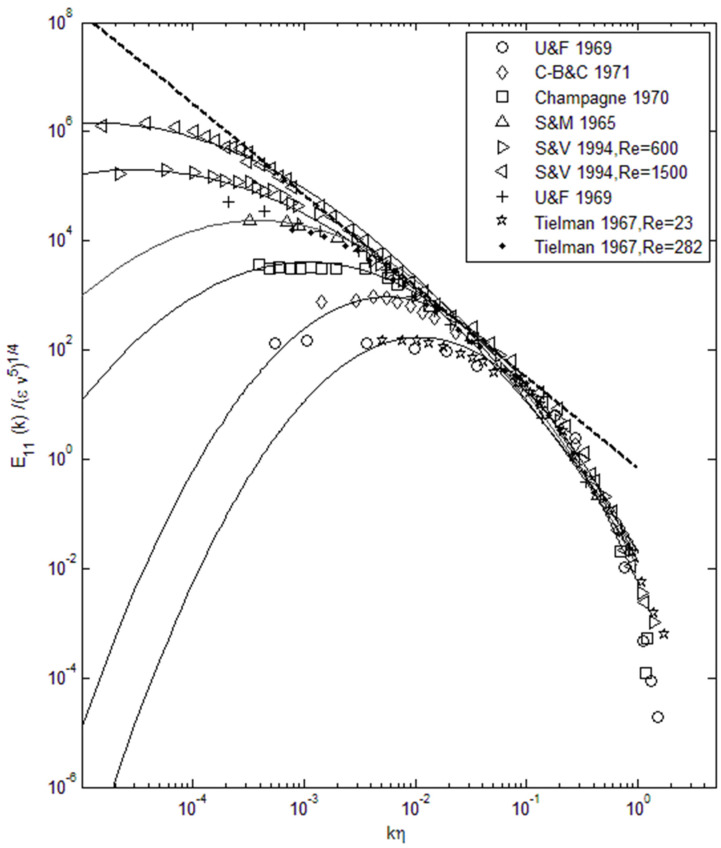
Lognormal turbulence energy spectra (**solid lines**) and experimental data. k^−5/3^ fit is plotted as a dashed line. Data (**symbols**) are for one-dimensional power spectra from the work shown in the legend [7,8,17,18,19].

Knowing the total energy and the width of the lognormal distribution allows us to construct the turbulent kinetic energy spectra over a wide range of Reynolds numbers, as shown in Figure 2. We can see in Figure 2 that energy spectra across a very wide range of energy and length scales are accurately reconstructed using the lognormal distribution function (plotted as lines) when compared with data (symbols). Kolmogorov’s k^−5/3^ scaling is also plotted (dashed line) for comparison, and we can see that for large Reynolds numbers this scaling is tangent to the lognormal distribution in the so-called inertial subrange. This is the region that contains a large portion of the total energy, and thus Kolmogorov scaling has been useful in prescribing the power spectra [16]. Figure 1 and Figure 2 show that quantitatively and qualitatively there is a close agreement between the lognormal distribution and observed turbulent energy spectra over almost the entire length scale range. The observed energy spectra end abruptly at the low wavenumber limit, corresponding to the length scale of the turbulence-generating object or process. Since the maximum entropy distribution has no knowledge of this process, it continues its downward path toward zero. To input the information concerning the turbulence generation, a truncated lognormal function can be used at the low wavelength limit. 

**Figure 3:**
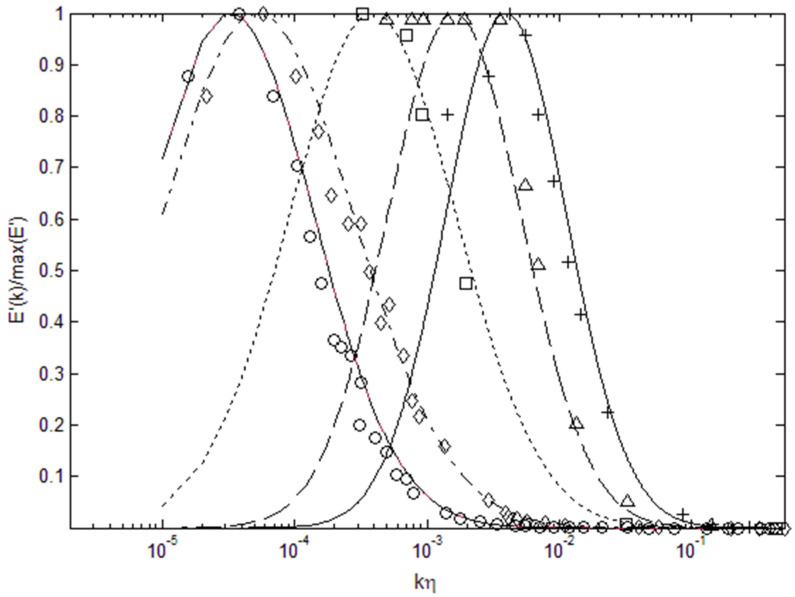
Lognormal distribution (normalized by the peak value) plotted in a semi-logarithmic scale. The data are from the same references as in Figure 2.

It is also interesting to plot the lognormal power spectra in a semi-logarithmic scale as in Figure 3, where there appears to be a shift in the spectra toward higher wavenumber; however, this is only due to nearly 5 orders of energy scales that have been normalized. It is only the wavenumber corresponding to the maximum energy which shifts toward smaller wavenumber as the Reynolds number increases. Additionally, the energy spectra tends to broaden relative to the Kolmogorov scale (ηk) when the Reynolds number increases. Thus, the energy spectra can be specified by the Reynolds number and energy scale of the turbulent flow. We can again see that the energy content is very small beyond the inertial subrange toward small length scales (large wavenumbers), which is why k^−5/3^ type of scaling is a reasonable approximation for power spectra in the energy-containing inertial range. 

**Figure 4:**
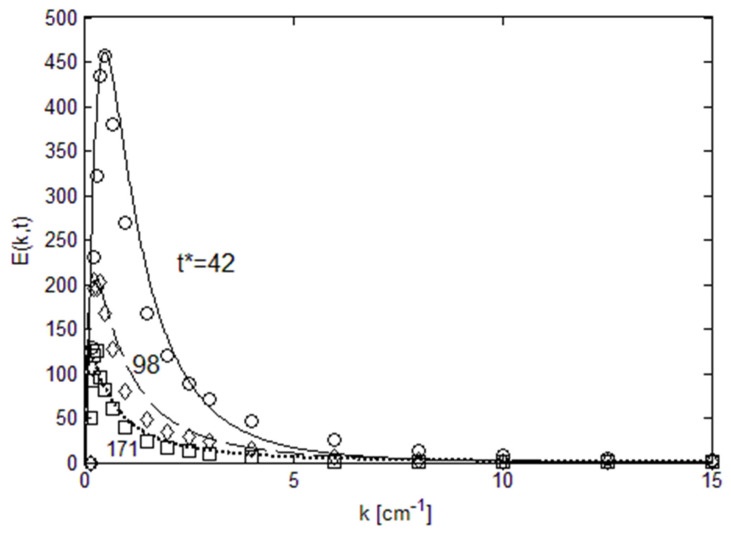
Temporal decay of energy spectrum. Data are from Comte-Bellot and Corrsin [7], and t* normalized time, t* = tU/M. Local Reynolds numbers are 71.6 (**circle**), 65.3 (**diamond**) and 60.7 (**square**).

We can also examine the change in the energy spectra in decaying turbulence. The experimental data of Comte-Bellot and Corrsin [7] illustrate this process, and the streamwise evolution of the power spectra is shown in Figure 4. The decay of turbulence energy is easily observable, where both the height and width of the distribution decreases with decreasing local Reynolds number. Yet, the lognormal shape of the power spectra is retained during the decay. Thus, lognormal distributions track both the shape and decaying magnitude of energy spectra. The information concerning the change in the height and width of the spectra can be used to reconstruct the local energy distributions using the Reynolds number as the determinant parameter. In Table 1, we show the maximum energy and width of the spectra (FWHM), both estimated from the data. Note that in Comte-Bellot and Corrsin [7] the data are given in dimensional units, and we retain the same units in Table 1. The data in Table 1 illustrate the parametric dependence of power spectra on the Reynolds number. Likewise, we extract the height and width information of the energy spectra from the data shown in Figure 2, and these are tabulated in Table 2. The data in Table 2 will be used later for parametric reconstruction of power spectra.

**Figure 5:**
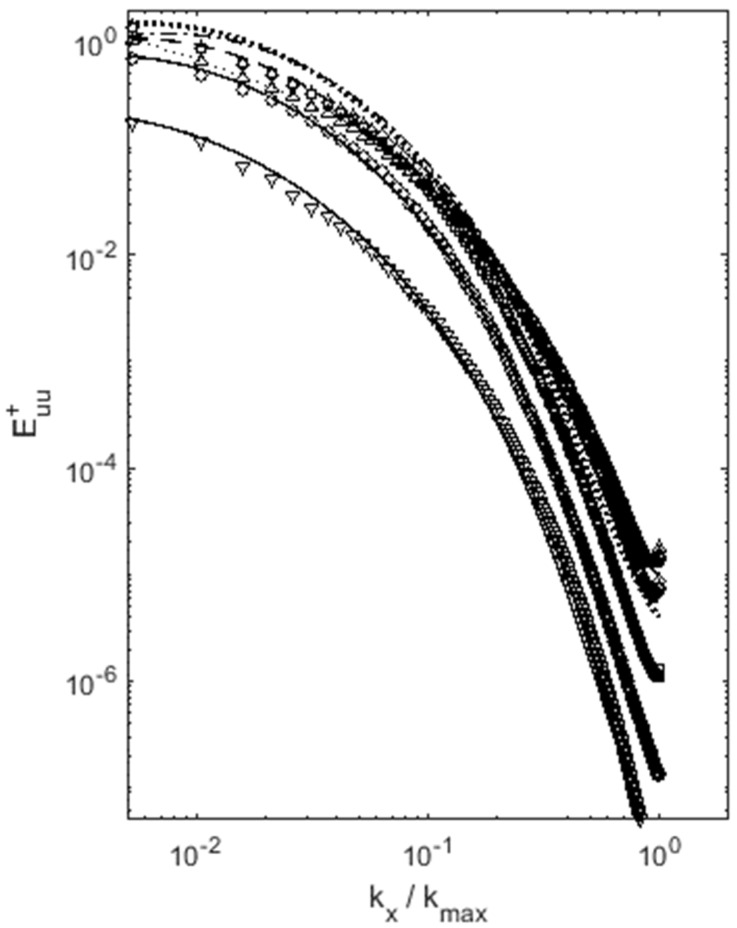
Turbulence energy spectra at various distances from the wall for channel flows, for Rel = 180 (**top left**), 395 (**top right**) and 590 (**bottom**). Lines are the lognormal distribution, compared with DNS data [21] with symbols (**circle**, y + = 5; **square**, y + = 10; **diamond**, y + = 20; **triangle up**, y + 30; **triangle down**, y + = 180 or 300.

The lognormal behavior of turbulence energy spectra is also evident in inhomogeneous flows, such as channel flows, as shown in Figure 5. Power spectra taken at various points in the channel all follow lognormal form to a remarkable degree. It appears that, if local equilibrium is achieved at high Reynolds numbers the state of maximum entropy exists locally and lognormal energy spectra are found even for inhomogeneous flows. This expands the applicability of current concept to inhomogeneous flows at sufficiently high Reynolds numbers. In addition, lognormal behavior is prevalent across nearly the entire range of scales, far beyond the so-called inertial range. 

Using the data in Table 2, we can also attempt to find a relationship between the Reynolds number and parameters that go into the lognormal function, so that we can reconstruct the energy spectrum based on the Reynolds number. To specify the height or the energy scale of the spectrum, a multiplicative factor, A, is used for the normalized lognormal function. The normalized lognormal function itself has two parameters, logarithmic mean, μ, and variance, σ. As shown in Figure 2, Figure 3 and Figure 4, we can see that μ decreases with increasing Re_λ_, while σ increases. Thus, we find a least-square fit to the following functions for these parameters using the data in Table 2.

Pre-exponential factor:

A = a_1_ × Re_λ_^2^ + a_2_ (1)

Logarithmic mean:

μ = b_1_ × Re_λ_^−3/2^ + b_2_ (2)

Variance:

σ = c_1_ × Re + c_2_ (3)

The form of these functions has been deduced from basic knowledge of length scale relationships to the Reynolds number [6]. There may be better functions for these parameters that reconstruct the energy spectra accurately at all the Reynolds numbers and physical configurations. Additionally, secondary parameters such as u′^2^, dissipation, kinematic viscosity, and/or other length scales may fine-tune the above functions. However, here we only demonstrate that turbulence energy spectra are recoverable through the lognormal distribution function by using simple functions for A, μ and σ, with the Reynolds number as the sole parameter.

**Figure 6:**
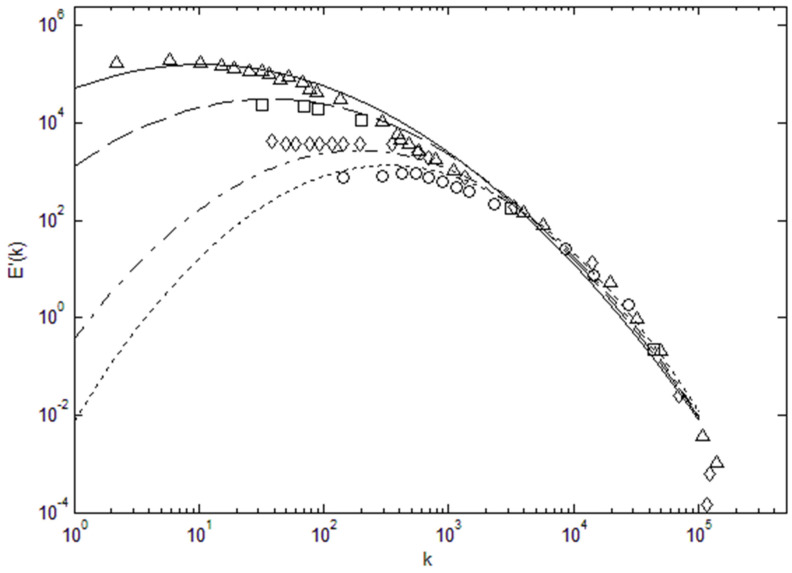
An illustration of power spectra reconstruction, using only the Reynolds number as a parameter in Equations (1)–(3). The data are from Comte-Bellot and Corrsin (**circle**) [7], Champagne, et al. (**diamond**) [8], Saddoughi and Veeravalli (**square**) [9], and Sanborn and Marshall (**triangle**) [18].

An example is shown in Figure 6, where we plot the reconstructed lognormal distributions using the parameters from Equations (1)–(3), and compare with some data. Note that we prefer to use the dimensional wavenumber, k, for this exercise. The energy scale is again E′(k) = E(k)/(εν^5^)^1/4^.

**Figure 7:**
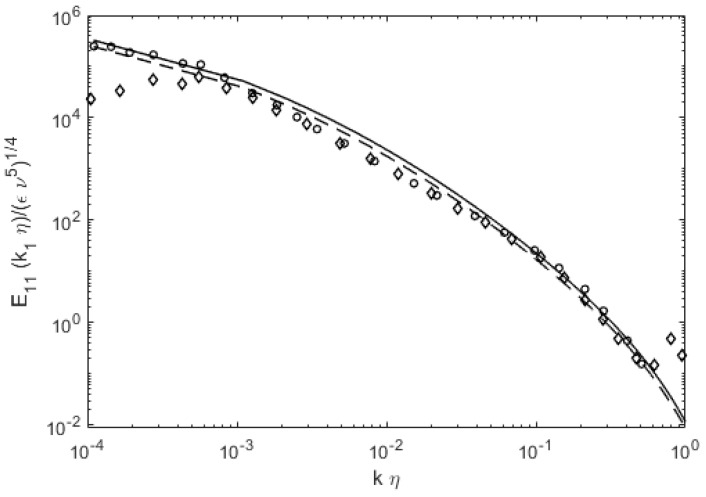
Comparison of the lognormal energy distribution with more recent data for E_11_ (**circle symbols**) and E_22_ (**diamond**), by Kang et al. [25]. Current theoretical lines are straight for E_11_ and dashed for E_22_.

There have been numerous works on finding the complete energy distributions in turbulence following the classical Kolmogorov theory [4], and also using the Navier–Stokes equation as the basis for the energy cascade, e.g., direct interaction approximation (DIA) [5]. A modified realizable version of the DIE theory is the so-called EDQNM (eddy-damped quasi-normal Markovian) [22]. EDQNM has a rather lengthy derivation process, and despite its mathematical sophistication reproduces the k^−5/3^ scaling in the inertial range extended by a triangular peak. There have also been function fits to estimate the power spectral form [23]. The current approach is based on the fundamental Second Law in the form of the maximum entropy principle, and avoids much of the mathematical complexities, to arrive at quite good agreements with data, as shown above. Figure 7 exhibits the comparison between the current lognormal form of the energy spectra with more recent data by Kang et al. [24]. We can see that the current lognormal distribution agrees quite well across a very large set of experimental data, and appears to have some universal applicability. As noted in the introduction, the lognormal energy spectra are arrived at either through a deductive process [2] or with a mathematical derivation (see Appendix A). In hindsight, the lognormal form could have been deduced since the energy spectra in turbulence is neither random or Gaussian, but asymmetric due to different physics involved at the low (energy generation) and high (viscous dissipation) wavenumbers and sectionally monotonic (it is highly improbable to have multiple peaks). Regardless of the route, lognormal distribution appears to be the natural selection for the energy distribution in turbulence due to asymmetric boundary conditions at extreme ends of the spectrum. In addition, the spectral parameters have intuitive dependence on the turbulence Reynolds number and length scales. It may be a good utilitarian example of the entropy science, toward a high-impact subject, turbulence, as the energy spectra have important implications in engineering and atmospheric science.

## 4. Conclusions

For turbulence energy spectra, a distribution function that maximizes entropy with the physical constraints is the lognormal function due to its asymmetrical descent to zero energy at the boundary lengths scales. Additionally, due to the improbability of multiple peaks the energy distribution is sectionally monotonic. The simplest distribution that satisfies these constraints is the lognormal distribution. The lognormal spectra are consistent with existing scaling laws such as Kolmogorov’s k^−5/3^ in the inertial range and k^4^ dependence in the large-eddy length scales. This approach makes it possible to reconstruct the turbulence energy spectra, using primarily the Reynolds number that determines the width and height of the lognormal distribution. There may be secondary parameters such as dissipation, kinematic viscosity, and lengths scales that can fine-tune the energy distribution, but the fundamental turbulence energy spectra exhibit lognormal behavior that can be prescribed by the Reynolds number, as stipulated by the known properties of the energy and length scales of turbulence. 

## Figures and Tables

**Table 1:** Parameters of the energy spectra for data of Comte-Bellot and Corrsin [7].

t*	Re_λ_	E_max_	E_total_	k(E_max_) [cm^−1^]	FWHM [cm^−1^]
42	71.6	461.2	774.6	0.058	1.24
98	65.3	212.6	342.8	0.12	0.98
171	60.7	123.9	174.9	0.22	0.84

**Table 2:** Parameters of the energy spectra for various data sets.

Re_λ_	E_max_/(εν^5^)^1/4^	(kη)_Emax_	Reference
37	130	0.0222	Comte-Bellot and Corrsin [7]
72	878	0.00439	Comte-Bellot and Corrsin [7]
308	53,100	0.0002084	Uberoi and Freymuth [17]
600	199,000	0.0000578	Saddoughi and Veeravalli [9]
850	312,900	0.000024	Coantic and Favre [20]
1500	1,446,000	0.0000179	Saddoughi and Veeravalli [9]

## Nomenclature

A	multiplicative constant (Equation (1))
a_1_, a_2_	constants for A (Equation (1))
b_1_, b_2_	constants for μ (Equation (2))
c_1_, c_2_	constants in σ (Equation (3))
C_1_, C_2_, C_3_	constants in the energy spectra equation (Equation (A4))
dV	spatial volume
e_o_	total energy at wavenumber k (Equation (A1))
E(k)	energy density at wavenumber k
E_11_, E_22_	energy density for the longitudinal (11) and lateral (22) components (Figure 7).
E′(k)	non-normalized energy density at wavenumber k
E^+^_uu_	energy density normalized by the friction velocity
F	objective function
FWHM	full width at half maximum
k	wavenumber
k_x_	wavenumber for the longitudinal spectra
*l*_e_	integral length scale
M	length scale
Re	Reynolds number
Re_λ_	Reynolds number based on the Taylor microscale
S	Shannon’s entropy
t	time
t*	tU/M normalized time
u′	turbulence fluctuating velocity
U	mean velocity
t	time interval
δt	time interval
ε	dissipation
η	Kolmogorov scale
μ	logarithmic mean in the lognormal distribution
ν	kinematic viscosity

## Appendix A

The energy distribution that maximizes the Shannon’s entropy under the physical constraints can be obtained using the Lagrange multiplier method [2,3]. Here, the principal constraint is that the turbulence conserves energy: the kinetic energy is dissipated by viscosity effect progressively at large wavenumbers [16].

(A1) $$u^{\prime 2}+\nu k^{2}u^{\prime 2}\delta t=e_{o}=constant$$

u′(k) the turbulent fluctuation velocity at a given wavenumber, k, while ν is the kinematic viscosity and δt some time interval. Equation (A1) states that turbulence energy density (on a unit-volume basis) integrated over some time interval δt is conserved. The available energy, u′^2^, is dissipated by the viscous term involving kinematic viscosity, ν [6], and thus Equation (A1) represents the conservation of energy in the k-space. The above constraint can be transposed into the energy distribution using the Lagrange multiplier method [2]. The first step is to write the objective function F so that

(A2) $$F=u^{\prime 2}+\nu k^{2}u^{\prime 2}\delta t-e_{o}$$

The most probable distribution function is found by maximizing logF, following the concept of Shannon’s entropy, S = FlogF [2]. We can see that S will achieve the maximum, when logF is at maximum or δ(logF) = 0. Using the Lagrange multiplier method, this leads to a distribution with an inverse exponential form [2].

(A3) $$E\left(k\right)dV=C_{1}exp\left\{-C_{2}u^{\prime 2}-C_{3}k^{2}u^{\prime 2}\right\}dV$$

C_1_, C_2_ and C_3_ (= C_2_ν) are so-called Lagrange multipliers, to be determined from other constraints. For example, C_1_ is determined by integrating E(k) to equal the total energy content in the distribution. Converting dV = d(k^−3^) to dk basis, we obtain the following energy distribution.

(A4) $$E\left(k\right)=\frac{C_{1}}{k^{4}}exp\left\{-C_{2}u^{\prime 2}-C_{3}k^{2}u^{\prime 2}\right\}$$

In Equation (A4), constants C_1_, C_2_, and C_3_ are determined from the constraints of the turbulence energy content, limiting length scales, and viscosity, respectively. C_1_ is the multiplier to the energy distribution, so that C_1_ increases for large turbulence energy content. For example, for large-scale high-speed flows, such as atmospheric flows, C_1_ will be a very large number (see Figure 2). C_2_ determines the width of the energy spectrum. The limiting length scales are the Kolmogorov dissipation length scale and the maximum length scale that exists in the flow, so that if either of these length scales are extended, then the energy spectrum will broaden. C_2_ is the parameter that reflects this width or the length scale effect. C_3_ is the viscous dissipation parameter, and depends strictly on the kinematic viscosity.

We still need the kinematic scaling for u′(k) in Equation (4). In Kolmogorov theory [6], u′ ~ k^−1/3^ is obtained in the inertial subrange. However, in the current maximum entropy formalism this is an unknown element or a lack of a piece of information. The maximum entropy principle gives the most probable energy distribution under the given physical constraints, but it does not produce unknown information. Thus, the missing pieces of information need to be supplied from observational data, and Equation (A4) provides a framework for testing various kinematic scaling for u′(k). Comparison with observational data can then be used to deduce the empirical form for u′(k) ~ (μ-log(k)). When this scaling is input in Equation (A4), we obtain a lognormal form in k.
